# Collagen/Gelatin/Hydroxyethyl Cellulose Composites Containing Microspheres Based on Collagen and Gelatin: Design and Evaluation

**DOI:** 10.3390/polym10040456

**Published:** 2018-04-19

**Authors:** Justyna Kozlowska, Natalia Stachowiak, Alina Sionkowska

**Affiliations:** Faculty of Chemistry, Nicolaus Copernicus University in Torun, Gagarina 7, 87-100 Torun, Poland; nat.sta@doktorant.umk.pl (N.S.); alinas@umk.pl (A.S.)

**Keywords:** collagen, gelatin, hydroxyethyl cellulose, microspheres, composites, crosslinking

## Abstract

The objective of this study was to develop three-dimensional collagen/gelatin/hydroxyethyl cellulose composites in combination with gelatin or collagen-gelatin loaded microspheres. Microspheres were prepared by an emulsification/crosslinking method. A 1-Ethyl*-*3-(3*-*dimethyl-aminopropyl)-carbodiimide (EDC) and *N*-hydroxysuccinimide (NHS) mixture were used as a crosslinking agent for the obtained materials. The structure of the materials was studied using scanning electron microscopy (SEM) and infrared spectroscopy. Moreover, a *Calendula officinalis* (pot marigold) flower extract release profile of the microsphere-loaded matrices was assessed in vitro. Additionally, porosity, density, stability, swelling and mechanical properties were tested. On the basis of SEM images, the microspheres exhibited a spherical shape and were irregularly dispersed in the polymer matrix. However, it was found that the addition of microparticles to obtained materials did not significantly change their microstructure. We observed a slight decrease in the swelling properties of matrices and an increase in values of Young’s modulus. Significantly, the addition of microspheres to the polymer matrices led to improved loading capacity of materials and release performance of *Calendula officinalis* flower extract. This makes the collagen/gelatin/hydroxyethyl cellulose composites containing microspheres a promising and suitable vehicle for biomedical, dermatological, or cosmetic applications.

## 1. Introduction

Microspheres are a type of microparticles in which the active substance is distributed in a polymer network*.* The size of microspheres ranges from 1 to 1000 µm. Naturally occurring polymers like collagen, gelatin, cellulose, chitosan, and alginate can be used to produce microspheres [1,2]. In recent years, porous matrices combined with micro/nano particles have been suggested as a new direction for drug delivery systems in biomedical or dermatological applications [3,4,5]. Del Mercato et al. fabricated biodegradable polyelectrolyte multilayer microcapsules, which were integrated into collagen matrices in the form of multifunctional three-dimensional scaffolds, a promising material for controlled delivery of bioactive molecules [3]. Collagen was also used for preparation of materials with magnetic properties. Magnetic particles with diameters of 5–10 nm and coated with chitosan were incorporated into a collagen 3D sponge. The results showed that these materials can be used as matrices for the delivery of compounds with magnetic properties [6]. Jiménez et al. successfully prepared collagen-gelatin microparticle-loaded collagen scaffolds using glutaraldehyde as a crosslinking agent. The results indicated that the obtained materials exhibited a porous structure with porosity greater than 90% and mean pore size of about 174 µm, which can be useful in tissue engineering to improve the skin regeneration process [7]. Lee et al. obtained 3D collagen/chitosan/glycosaminoglycan scaffolds in combination with transforming growth factor-beta 1 (TGF-β1)-loaded chitosan microspheres, and their results expanded the feasibility of combinative strategies for controlled drug release and tissue-engineered cartilage formations [8].

Collagen is a fibrous protein found in connective tissue. It represents nearly 30% of total proteins in animal organisms [9]. The molecular structure of collagen consists of three polypeptide chains of similar lengths in helical conformation winding around each other [10]. A characteristic of collagen is the presence of specific repeating amino acid sequences, glycine-X-Y, where X and Y are frequently proline and hydroxyproline [11]. Currently, 29 different types of collagen (Type I-XXIX) have been identified [12]. However, due to the fact that type I collagen plays a structural role, it also contributes to the molecular architecture, shape, and mechanical properties of tissues [13]. Collagen, because of its non-toxicity, non-antigenicity, and biocompatibility, is an ideal biomaterial for tissue engineering, wound dressing applications, and drug delivery systems [14,15]. However, susceptibility to enzymatic in vivo degradation rates and low mechanical strength have restricted the application of collagen. To enhance the physical properties of collagen-based materials, it is necessary to stabilize the collagen structure. These materials are frequently stabilized by crosslinking modification [16,17,18]. A 1-Ethyl*-*3-(3*-*dimethyl-aminopropyl)-carbodiimide (EDC) and *N*-hydroxysuccinimide (NHS) mixture has become a popular crosslinking reagent for collagen-based biomaterials [16,19,20,21].

Conducting thermal denaturation of collagen results in the receipt of gelatin. Commercial gelatin is primarily produced from bovine or porcine skin and bones by partial acid hydrolysis (type A) or partial alkaline hydrolysis (type B). It is characterized by different isoelectric points, ranging from 7.0 to 9.0 for type A and from 4.7 to 5.3 for type B. A special feature of gelatin is the ability to form gels [8,22,23]. Gelatin is available at a relatively low cost and it possesses some useful properties, such as biodegradability and non-immunogenicity [24]. Gelatin, as a water-soluble natural polymer, is widely used in food, pharmaceutical, cosmetic, and photographic applications [25]. Gelatin has also been used in sensors and the biomedical field [11,26,27,28,29]. Adhirajan et al. modified gelatin microspheres and after loading them with doxycycline, they were impregnated in a reconstituted collagen scaffold as a novel wound dressing [24]. DeFail et al. used gelatin to prepare a controlled delivery system of doxorubicin to maintain local levels of the drug wherein the drug’s release was controlled by the incorporation of poly(lactic-*co*-glycolic acid) microspheres into gelatin constructs [30].

Cellulose, a natural polymer widely distributed in nature, is a linear polysaccharide composed of 1,4-β-d-glucopyranosyl units [31]. This biopolymer is insoluble in both cold and hot water and in organic solvents. For this reason, cellulose modifications are made to attain water-soluble derivatives, thus broadening the scope of its applications [32]. The cellulose ethers obtained by etherification of alcohol groups are commonly used for this purpose. Significant reactions occur in amorphous areas or on the surface of the fibers. This usually leads to a reduction in the average molecular weight of the cellulose [31,33]. Hydroxyethyl cellulose is one of the most important commercial soluble cellulose derivatives obtained through the reaction of ethylene oxide on alkali cellulose [34]. Hydroxyethyl cellulose exhibits features such as good thickening, suspension, dispersion, emulsification, and film-forming. Hydroxyethyl cellulose is utilized particularly in the chemical industry and in everyday life as an emulsifier, stabilizer, thickener and film-former in cosmetic formulations due to the nonionic characteristics of unreactive ions [35].

*Calendula officinalis* is a medicinal plant also known as pot marigold. Its flower extract consists of carotenoids, terpenoids, flavonoids, quinines, coumarins, and other constituents and has been reported to exhibit anti-cancer activity, anti-inflammatory effects, and antimicrobial properties. The flower extract of this plant has been used for the treatment of burns, ulcers, skin inflammations, eczema, and wounds [36,37]. The pharmacological activity of *Calendula officinalis* flower extract could be enhanced by its incorporation into a polymeric matrix that controls the release of active compounds over time.

In this study, porous matrices made of collagen, gelatin, and hydroxyethyl cellulose were prepared via the freeze-drying method. The microspheres based on gelatin or collagen-gelatin mixtures were incorporated into the porous matrices without significantly modifying their microstructure. An EDC/NHS mixture was used as a crosslinking agent for the materials. The morphology and size of the microparticles, as well as the morphology, porosity, swelling properties, mechanical properties, and stability of microsphere-loaded materials were measured. This novel delivery system was tested to control the release of *Calendula officinalis* flower extract.

## 2. Materials and Methods

### 2.1. Materials

Collagen (col) type I was obtained in our laboratory from fish scales of *Esox lucius* [38]. Gelatin type A (gel), hydroxyethyl cellulose (hec), Span 85, 1-ethyl-3(3-dimethylaminopropyl) carbodiimide (EDC), *N*-hydroxysuccinimide (NHS), Folin-Ciocalteu reagent, and gallic acid were purchased from Sigma-Aldrich (Poznan, Poland). Hydrochloric acid, acetic acid, sodium biphosphate anhydrous, sodium phosphate, sodium acetate anhydrous, and sodium chloride were obtained from Avantor Performance Materials Poland S.A. (Gliwice, Poland). Ethyl alcohol, paraffin oil, disodium edetate dihydrate, and sodium carbonate were supplied by the Stanlab (Lublin, Poland). The hydroglycolic *Calendula officinalis* flower extract was acquired from Provital S.A. (Barcelona, Spain).

### 2.2. Production of Gelatin and Collagen-Gelatin Microspheres

Collagen-gelatin (col-gel) microspheres were prepared by a water-in-oil emulsion described by Kawai et al., with modifications [39]. Gelatin type A (0.5 g) was added to a 1% *w*/*v* collagen suspension (2.5 g). The mixture was heated at 40 °C for 30 min. After that time, 40 mL of paraffin oil containing 1% Span 85 was added to the mixture and homogenized (3000 rpm, 20 min) using a T25 digital ULTRA-TURRAX disperser (IKA Werke, Staufen, Germany). The obtained water-in-oil emulsion was magnetically stirred and microspheres were crosslinked in the presence of 50 mM EDC and 25 mM NHS at room temperature for 4 h. The crosslinked microspheres were separated by centrifugation (10,000 rpm, Eppendorf AG Centrifuge 5805R, Hamburg, Germany) for 10 min, and washed four times with 96% ethanol. The product was frozen (−20 °C) and lyophilized (−55 °C, 5 Pa, 24 h) using an ALPHA 1-2 LD plus lyophilizator (Martin Christ, Osterode am Harz, Germany). The gelatin microspheres (gel) were fabricated from 20% *w*/*v* aqueous gelatin solution, heated at 40 °C for 30 min following the procedure described for col-gel microspheres but without the addition of 1% *w*/*v* collagen suspension.

### 2.3. Production of Collagen-Gelatin Matrices Incorporating Gel and Col-Gel Microspheres

To prepare microsphere-loaded matrices, gel (0.15 g) or col-gel (0.15 g) microspheres were mixed with 15 mL collagen suspension (0.5% *w*/*v*), 15 mL aqueous gelatin type A solution (0.5% *w*/*v*), and hydroxyethyl cellulose (0.15 g) and magnetically stirred for 30 min. The mixture was then frozen (−20 °C) and lyophilized (−55 °C, 5 Pa, 24 h). After lyophilization, matrices were crosslinked using a crosslinking mixture. The chemical modification of the samples was carried out in 96% ethanol in the presence of 50 mM EDC and 25 mM NHS at room temperature for 4 h. After this time, the matrices were washed with 0.1 M Na_2_HPO_4_ (washing was done for 2 h, changing the solution after 1 h). To remove the residue of the crosslinking mixture, the samples were washed 4 times with distilled water (washing was performed for 2 h, changing the water every 30 min). The crosslinked matrices were frozen (−20 °C) and lyophilized (5 Pa, −55 °C, 48 h). Collagen/gelatin/hydroxyethyl cellulose matrices incorporating gelatin microspheres were named col/gel/hec (gel) and matrices incorporating collagen-gelatin microspheres were named col/gel/hec (col-gel). A matrix without microspheres was used as a control and was named col/gel/hec.

### 2.4. Characterization of the Composites

#### 2.4.1. Scanning Electron Microscopy (SEM)

Scanning electron microscopy (SEM) imaging was performed using the Quanta 3D FEG scanning electron microscope manufactured by Quorum Technologies (Lewes, UK). The matrices and crosslinked microspheres were subjected to SEM analysis to examine the surface structure of the obtained materials. Prior to the analysis, a thin layer of gold and palladium was sprayed onto the surface of the samples (SC7620 Mini Sputter Coater/Glow Discharge System, Quorum Technologies).

#### 2.4.2. Attenuated Total Reflection Infrared Spectroscopy (ATR-FTIR)

The structure of the samples was evaluated by attenuated total reflection infrared spectroscopy (ATR-FTIR) using a Nicolet iS10 FTIR spectrophotometer (Thermo Scientific, Waltham, MA, USA) equipped in an attenuated total reflection (ATR) device. All spectra were recorded in absorption mode at 4 cm^−1^ intervals and 64 scans.

#### 2.4.3. Porosity and Density Measurement

The density and porosity of the obtained matrices were measured by liquid displacement [6,9]. The liquid used in this study was isopropanol because it penetrated easily into the pores and did not cause shrinkage or swelling as a nonsolvent of the matrix-forming polymers. A sample with a known weight (*W*) was immersed in a graduated cylinder in a known volume of isopropanol (*V*_1_) for 5 min. The total volume of isopropanol in the cylinder and the isopropanol-impregnated matrix was *V*_2_. The isopropanol-impregnated matrix was removed from the cylinder and the residual isopropanol volume was recorded (*V*_3_). Each sample was measured in triplicate. The density of the porous samples (*d*) and the porosity of the matrices (Є) are expressed as follows:

$$d=\frac{W}{V_{2}-V_{3}}$$

$$Є\;\left(\%\right)=\left[\frac{\left(V_{1}-V_{3}\right)}{\left(V_{2}-V_{3}\right)}\right]\;\times 100$$

#### 2.4.4. Swelling Tests

A piece of each dried porous matrix was weighed (*W*_w_) and then immersed in 3 mL of phosphate buffer saline (PBS, pH = 5.7) for 15 min, 30 min, 1 h and 2 h. After each time period, the matrices were taken out of the solution. After removal from the PBS solution, the samples were weighed (*W*_d_). Each value was averaged from three parallel measurements. The swelling ratio of the matrix was defined as the ratio of the weight increase (*W*_w_ − *W*_d_) to the initial weight (*W*_d_), according to following equation:

$$Swelling\;ratio\;\left(\%\right)=\left[\frac{\left(W_{w}-W_{d}\right)}{W_{d}}\right]\times 100$$

#### 2.4.5. Dissolution of Matrices

Crosslinked matrices were weighed prior to the dissolution study (*W*_b_). The samples were immersed in 3 mL PBS (pH = 5.7) at room temperature for 1, 2, 3, and 4 weeks. After each time period, they were removed from the PBS buffer, rinsed with deionized water three times, frozen, lyophilized and weighed (*W*_a_). The percentage weight loss was calculated from the dry weight before and after being immersed in PBS.

$$Weight\;loss\;\left(\%\right)=\left[\frac{\left(W_{b}-W_{a}\right)}{W_{b}}\right]\times 100$$

The experiment was carried out for three samples and the average value was recorded.

#### 2.4.6. Mechanical Properties

Cylindrical samples with a diameter of ~16 mm and a height of ~21 mm were produced for mechanical testing by placing each of the col/gel/hec and microspheres mixtures into polystyrene cylindrical containers, freezing, and then freeze-drying them. All testing was carried out using a mechanical testing machine (Z.05, Zwick/Roell, Ulm, Germany) at room temperature. Dry samples as well as samples soaked in the PBS buffer at pH 5.7 were used for the analysis. Prior to the measurements, the samples were carefully measured (diameter and height). Next, samples were placed between the compression plates of a computer-controlled testing machine and they were pressed with the cross-head speed set at 2 mm·min^−1^. The results were recorded using the testXpert II computer program (Zwick/Roell, Ulm, Germany). The compressive modulus (Young’s modulus, *E*_c_) was calculated from the slope of the stress-strain curve in the linear region (strain from 2% to 5%). The presented values are the average values calculated from five measurements for each type of matrix.

### 2.5. Incorporation of Calendula Officinalis Extract into the Matrices

Fragments of prepared matrices (1 cm × 1 cm) were immersed in 3 mL of 2% *v*/*v* commercial *Calendula officinalis* hydroglycolic extract for 24 h (*n* = 3). Then, they were vacuum-degasified (15 min) and incubated (37 °C, 24 h). The non-absorbed extract was removed and the surfaces of the matrices were washed with distilled water to eliminate the excess extract. The material was frozen (−20 °C) and lyophilized.

### 2.6. Loading Capacity of Matrices

*Calendula officinalis* extract-loaded fragments of matrices (1 cm × 1 cm) were weighed and placed in 2 mL of 1 M NaOH for 1 h. The resulting suspension was centrifuged (10,000 rpm, 5 min) and the supernatant solution was collected. The polyphenolic compounds were quantified by using the Folin-Ciocalteu test [40]. Samples with the extract (20 μL) were mixed with 1.58 mL distilled water and 100 µL Folin-Ciocalteu reagent. After 4 min, 300 mL of saturated Na_2_CO_3_ solution was added. The prepared mixtures were incubated (40 °C, 30 min), until a characteristic blue color was obtained. The absorbance was measured at 725 nm using a UV-Vis spectrophotometer (UV-1800, Shimadzu, Kyoto, Japan). The values were marked on the calibration curve for the standard solution (gallic acid) in the concentration range 0–0.50 mg/mL (*R*^2^ = 0.9945). The results presented are the average of measurements made for three samples of each type of matrix.

### 2.7. In Vitro Release

*Calendula officinalis* extract-loaded fragments of the matrices (1 cm × 1 cm) were placed in a polystyrene 12-well plate and 2 mL of 0.2 M acetate buffer (pH = 5.7) was added to each sample. The plate with the samples was incubated at 37 °C for 7 days. The solution was collected after 1, 2, 3, 4, and 7 days, each time adding acetate buffer stored at 37 °C. The obtained samples were frozen (−20 °C) and after 7 days, the content of the polyphenolic compounds was determined using the Folin-Ciocalteu test [40]. The collected samples (20 μL) were mixed with 1.58 mL distilled water and 100 µL Folin-Ciocalteu reagent. After 4 min, 300 mL of saturated Na_2_CO_3_ solution was added. The prepared mixtures were incubated (40 °C, 30 min), until a characteristic blue color was obtained. The absorbance was measured at 725 nm using a UV-Vis spectrophotometer (UV-1800, Shimadzu). The experimental calibration curve for the standard solution (gallic acid), prepared previously for loading capacity was used. The measurements were carried out for three samples of each type of matrix and the average value was recorded.

## 3. Results and Discussion

### 3.1. Structure of Prepared Materials, Porosity and Density

**Figure 1:**
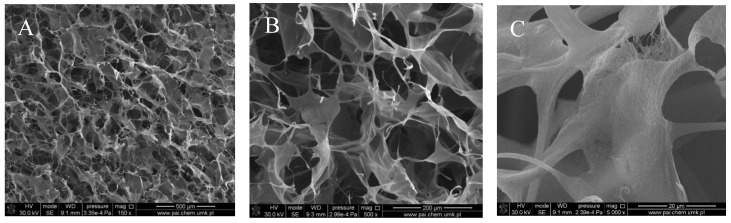
Scanning electron microscopy (SEM) images of the collagen/gelatin/hydroxyethyl cellulose (col/hec/gel) matrix without microspheres: (**A**) magnification ×150; (**B**) magnification ×500; (**C**) magnification ×1000.

**Figure 2:**
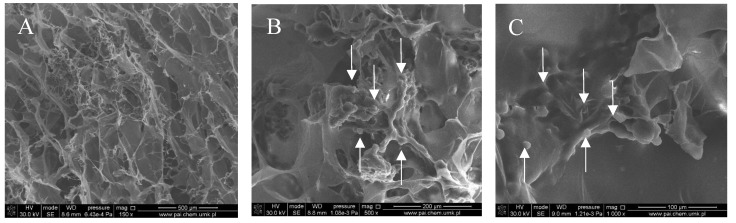
SEM images of the col/gel/hec matrix containing gelatin microspheres. Microspheres are indicated by arrows: (**A**) magnification ×150; (**B**) magnification ×500; (**C**) magnification ×1000.

**Figure 3:**
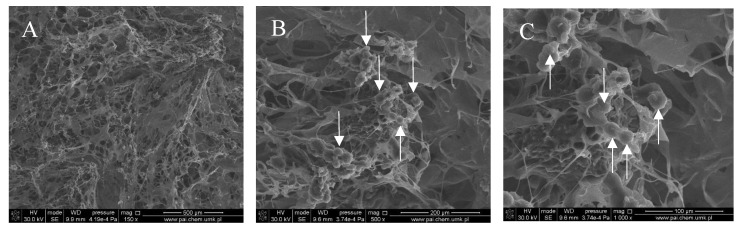
SEM images of the col/gel/hec matrix containing collagen-gelatin microspheres. Microspheres are indicated by arrows: (**A**) magnification ×150; (**B**) magnification ×500; (**C**) magnification ×1000.

**Figure 4:**
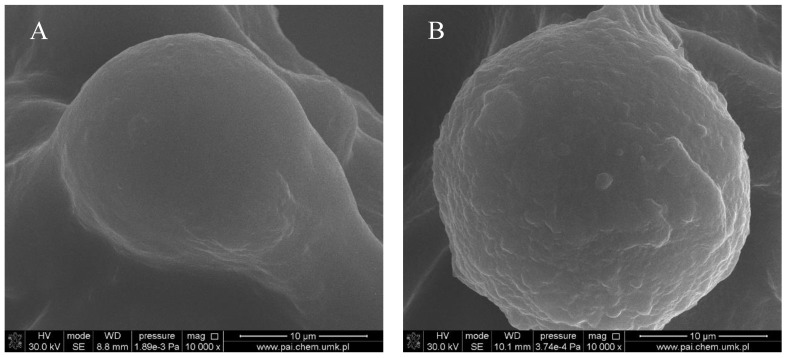
Microsphere accommodated within a col/gel/hec matrix pore: (**A**) gelatin microsphere; (**B**) collagen-gelatin microsphere.

Figure 1, Figure 2, Figure 3 and Figure 4 show images of the obtained 3D matrices. The structure of the prepared col/gel/hec matrices had irregular macropores and excellent interconnectivity. The presented pictures illustrate an evident difference between the control sample (Figure 1) and the microsphere-loaded matrices (Figure 2 and Figure 3). The microspheres can be observed in the form of round places non-uniformly accommodated onto the porous surface of the matrix. The morphological observations revealed that both the gel and col-gel microparticles possessed a spherical shape and smooth surface (Figure 4). The diameter of the gel microspheres was about 21.1 ± 7.3 µm and was slightly smaller than that of the col-gel microspheres. Gelatin is a denatured fibrous protein derived from collagen by partial thermal hydrolysis. Rössler et al. have indicated that the denaturation grade of collagen is the most important factor in influencing the size of microparticles [41]. Total denaturation of collagen results in degradation and subsequently yields smaller microparticles. This suggests that the decrease in size of collagen-gelatin microspheres is caused by the addition of gelatin. Biopolymer spherical microparticles are arranged in an irregular manner, in some places forming agglomerates.

The results of porosity and density measurements are shown in Table 1.

The porosity of all composites was more than 79%. The porosity of the pure col/gel/hec matrix was 86.0 ± 2.79% and with the addition of microspheres, we observed the significant decrease of porosity to 79.3% and 81% for col/gel/hec (gel) and col/gel/hec (col-gel), respectively. It seemed that the decrease of porosity was caused by the decrease in an amount of ice crystals when the collagen/gelatin/hydroxyethyl cellulose solution containing microspheres was frozen. Materials with incorporated microspheres had a higher degree of density 0.014 g/cm^3^ (col/gel/hec (gel)) and 0.017 g/cm^3^ (col/gel/hec (col-gel)) in comparison with materials without microspheres (*d* = 0.012 g/cm^3^).

### 3.2. ATR-FTIR Spectroscopy Results

**Figure 5:**
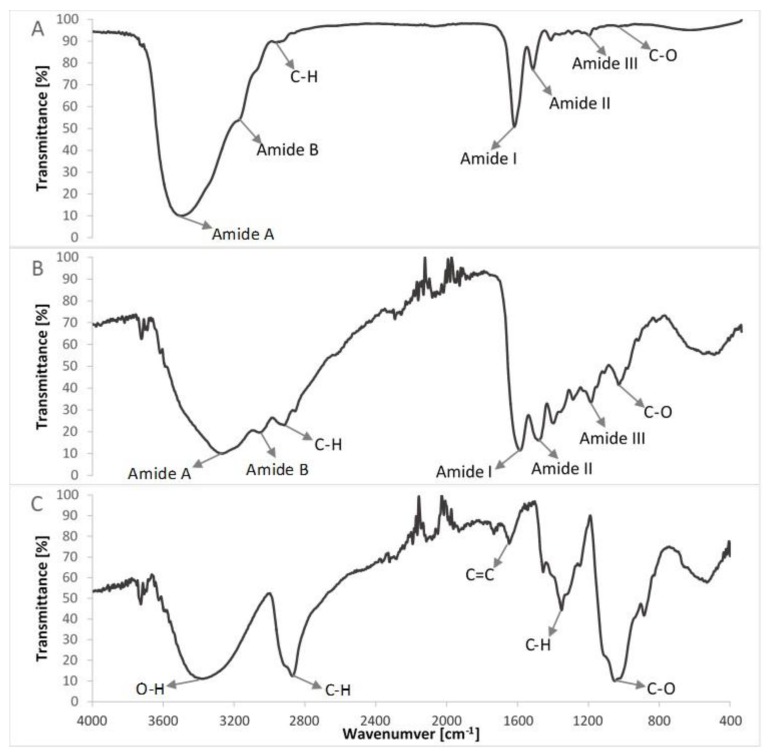
Comparison of FTIR spectra of non-crosslinked compounds of studied composites (**A**) lyophilized collagen (extracted from the scales of northern pike (*Esox lucius*); (**B**) gelatin; (**C**) hydroxyethyl cellulose.

**Figure 6:**
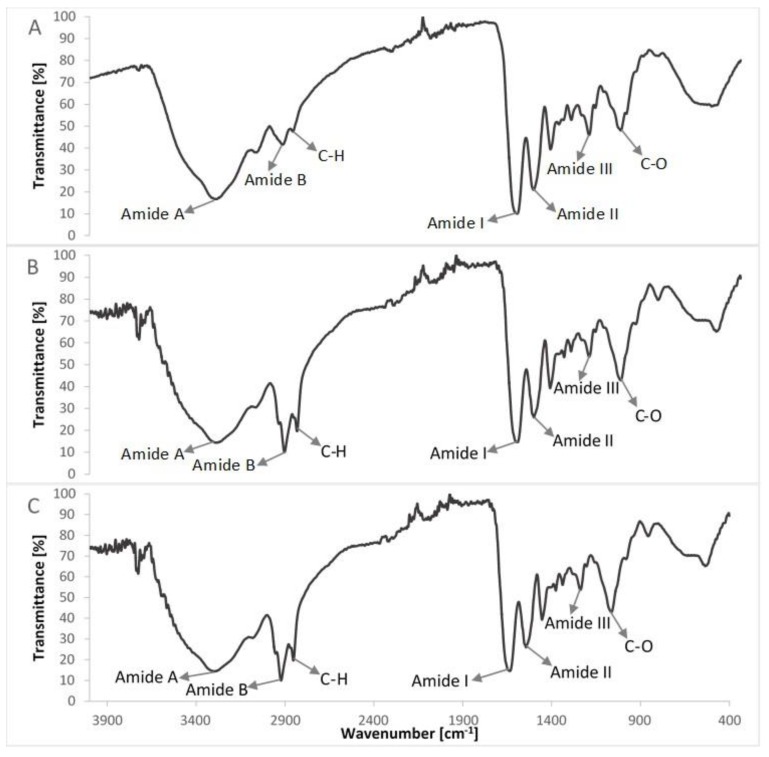
Comparison of FTIR spectra of col/gel/hec composites with and without microspheres in the structure: (**A**) col/gel/hec; (**B**) col/gel/hec (gel); (**C**) col/gel/hec (col-gel).

FTIR spectra of compounds of the col/gel/hec matrices are shown in Figure 5 and the spectra of collagen/gelatin/hydroxyethyl cellulose-based matrices are shown in Figure 6. The position of the main band in FTIR spectra of studied samples is presented in Table 2.

The FTIR spectra of pure collagen and gelatin matrices (Figure 5A,B, Table 2) had five characteristic absorption bands: amide A (corresponding to the stretching bonds of N–H coupled with hydrogen bonding), amide B (corresponding to the stretching vibrations of N–H in the Fermi resonance with the first overtone of amide II), amide I (representing the stretching of C=O bonds), amide II (bands related to the coupled bending bonds of N–H and stretching bonds of C–N) and amide III (corresponding to the stretching bonds of C–H and bending bonds of N–H) [38,42]. In the case of the spectrum of hydroxyethyl cellulose (Figure 5C), the hydroxyl group stretching vibration was observed at 3370 cm^−1^, whereas that for the C–H bond stretching vibration was observed at 2867 cm^−1^. The band at 1047 cm^−1^ indicated C–O stretching vibration [35,43].

The IR spectra of prepared composite materials were characterized by absorption bands arising from collagen, gelatin and hydroxyethyl cellulose. The positions of amide bands for composite col/gel/hec matrices were nearly unchanged when compared with the position of amide bands for pure collagen and gelatin. We observed a shift of position for all the amide bands to frequencies between positions on the FTIR spectra of pure collagen and gelatin. The shift of amide bands after mixing of collagen, gelatin, and hydroxyethyl cellulose suggested a new interaction between these compounds of matrices. Usually, the shift of bands in IR spectra is due to interactions between biopolymers by hydrogen bonds or electrostatic interactions [44]. The slight shifts of the position of amide bands may suggest that the secondary structure of collagen is not destroyed after a crosslinking reaction.

As one can see in Figure 6 and Table 2, the addition of microspheres to collagen/gelatin/hydroxyethyl cellulose matrices did not change the position of the main bands in IR spectra compared with the spectrum of col/gel/hec without microspheres.

### 3.3. Swelling Tests

**Figure 7:**
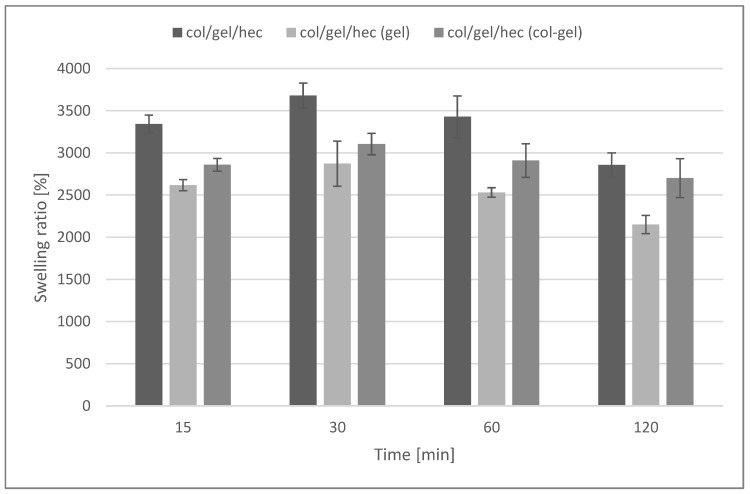
Swelling percentage of col/gel/hec matrices with and without microspheres.

Figure 7 shows the swelling ratios of collagen/gelatin/hydroxyethyl cellulose composites immersed in phosphate buffer saline (pH = 5.7) during 2 h of the test. The maximum degree of swelling was noted after 30 min of incubation in the buffer. After that time, matrices without the addition of microspheres absorbed the largest amount of PBS (3680 ± 148%). For materials containing microspheres, a decrease in the swelling degree was observed (swelling ratio after 30 min was about 2872 ± 267% and 3105 ± 127% for matrices containing gelatin and collagen-gelatin microspheres, respectively).

The high swelling properties are characteristic for hydrophilic polymers (such as collagen, gelatin, and hydroxyethyl cellulose), as well as for materials with porous structures [45]. The addition of microparticles decreased swelling properties and was probably related to the reduction of the porosity of col/gel/hec matrices after the addition of microparticles. However, samples containing microspheres were still characterized by a high swelling ability.

### 3.4. Dissolution of Matrices

**Figure 8:**
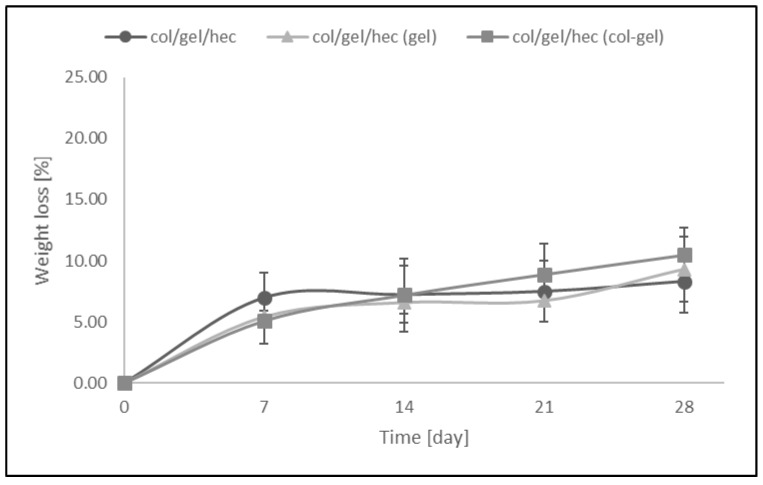
The values of weight loss during degradation of col/gel/hec-based matrices after immersed in phosphate buffer saline (PBS).

Figure 8 shows the weight loss (%) of collagen/gelatin/hydroxyethyl cellulose-based materials over the time when the samples were soaked in PBS (pH = 5.7). The resistance to dissolution of samples was investigated every 7 days up to 4 weeks. The results revealed that the greatest loss of mass occurred within the first 7 days of a sample’s incubation in PBS buffer (the weight loss of samples was about 5–7% in this time). We did not observe significant differences between the degradation rates of matrices containing microspheres and samples without them. The addition of the microparticles into polymer matrices had no influence on resistance to degradation of the materials. After 28 days of being soaked, the weight loss of microsphere-loaded matrices was only about 8–10.5%.

### 3.5. Mechanical Properties

The mechanical properties of studied samples were measured under different conditions: in room-dry conditions and in PBS for swollen samples soaked during testing. Young’s modulus was measured and the results are shown in Table 3.

Based on the obtained results for dry samples, we observed that the incorporation of microspheres into col/gel/hec matrices did not have a significant effect on the mechanical properties of the studied materials. However, in hydrated conditions, we observed significant changes in values of Young’s modulus. After soaking the samples in the PBS buffer, we noted a significant decrease in values of Young’s modulus. Very high swelling capacity of its scaffolds led to the decrease of its stiffness. The decrease of Young’s modulus of the matrices after they had been placed in wet conditions was probably due to hydration, as water acts as a plasticizer. A drop in stiffness was observed upon hydration, probably caused by the weakening of hydrogen bonds within the molecular structure of the collagen. Moreover, one can see that with the addition of microparticles into col/gel/hec matrices, the values of Young’s modulus (and thus the stiffness of the samples) also increased and this is probably related to the increase of the density of these samples (Table 1).

### 3.6. Loading Capacity of Matrices

**Figure 9:**
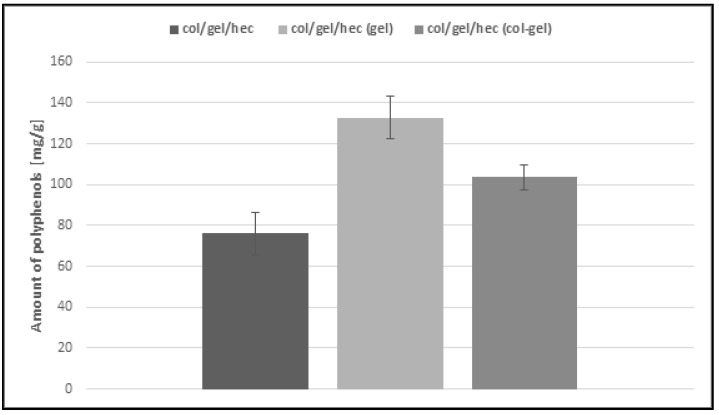
Loading capacity of col/gel/hec-based matrices.

The *Calendula officinalis* flower extract was loaded over 24 h to the obtained collagen/gelatin/hydroxyethyl cellulose-based matrices. As one can see in Figure 9, the pot marigold flower extract loading is more efficient in the case of microsphere-loaded matrices than those not containing microspheres. This behavior probably stemmed from the ability of the microparticles to swell and retain greater amounts of extract than the unmodified collagen/gelatin/hydroxyethyl cellulose matrices. Another important observation is that the effectiveness of loading extract in the case of gelatin microsphere-loaded matrices was the highest (about 133 mg/g) and it was two times more than that of the sample without microspheres. It seems that it is a feature conferred by gelatin, which usually displays higher swelling capacity than collagen [7,46]. Col/gel/hec (gel) matrices contained the largest amount of gelatin in the structure and this in turn increased the loading capacity of materials.

### 3.7. In Vitro Release

**Figure 10:**
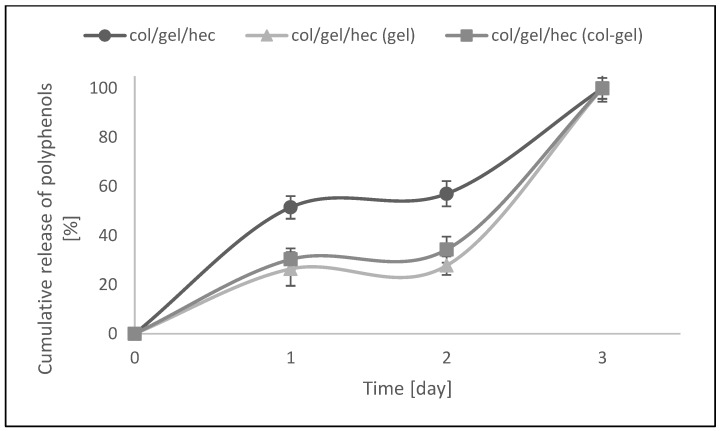
In vitro release assays of col/gel/hec matrices with and without microspheres.

The modification of col/gel/hec matrices by the addition of microspheres was also beneficial to controlling the release of the pot marigold flower extract. Figure 10 shows the *Calendula officinalis* flower extract release profile from collagen/gelatin/hydroxyethyl cellulose-based materials in acetate buffer (pH 5.7) at 37 °C. There was a burst release at an initial stage, followed by a sustained release of *Calendula officinalis* flower extract. The faster and higher burst release of extract might be due to the amount of entrapped extract bound to the surface of the collagen/gelatin/hydroxyethyl cellulose matrix [47].

As one can see in Figure 10, the active substance incorporated into the control sample was released two times faster than that in the microparticle-loaded matrices. After the first day, about 51.5% of the active substance was released. For comparison, in the case of microsphere-loaded matrices, this value after the first day of incubation was about 26%. Furthermore, the second phase (from day 1 to day 2) was characterized by a slower release rate and can be associated with the time required for polyphenol diffusion from the collagen/gelatin/hydroxyethyl cellulose matrix and microparticles to the release medium. In col/gel/hec composite matrices, microparticles served as extract reservoirs, which significantly affected the release profile of the active substance. Additionally, it was noted that the greater concentration of loaded polyphenols in the matrices, the slower the release rate. With the addition of microspheres, we observed the significant decrease of porosity and this in turn hindered diffusion phenomena, especially from the inner portions of the construct [7]. Col/gel/hec matrices containing gel microspheres had the lowest degree of porosity and the lowest cumulative release of polyphenols. In the next phase (from day 2 to day 3) the release of active substance was faster for all samples. This might be due to the fact that the gelatin and collagen-gelatin microspheres may swell in the release medium and the entrapped extract might gradually dissolve and diffuse through the swollen polymeric matrix to the release medium [47].

Results indicated that complete release of pot marigold extract from all studied samples was observed after 3 days of incubation at 37 °C in acetate buffer.

## 4. Conclusions

Crosslinked gelatin and collagen-gelatin microspheres with a spherical shape and smooth surface were synthesized with particle sizes ranging ~21–26 µm. Porous three-dimensional collagen/gelatin/hydroxyethyl cellulose matrices containing microspheres were fabricated and characterized. This system based on the incorporation of microspheres into col/gel/hec matrices had high porosity and swelling properties as well as high resistance to dissolution and better mechanical properties compared to matrices not containing microspheres in the structure. The modification of a polymer matrix by the incorporation of microparticles increased loading capacity and release performance, making it a promising material in biomedical or cosmetic applications. This was especially true in the case of samples with the addition of gelatin microspheres, which were characterized by high porosity, the highest loading capacity of *Calendula officinalis* flower extract, and the least cumulative release of polyphenols.

## Figures and Tables

**Table 1:** Porosity (Є) and density (*d*) of the collagen/gelatin/hydroxyethyl cellulose (col/gel/hec) matrices.

Sample	Є (%)	*d* (g/mL)
col/gel/hec	86.0 ± 2.79	0.012 ± 0.0019
col/gel/hec (gel)	79.3 ± 1.28	0.014 ± 0.0020
col/gel/hec (col-gel)	81.0 ± 3.21	0.017 ± 0.0017

**Table 2:** The position of main bands (cm^−1^) in FTIR spectra of non-crosslinked lyophilized collagen and gelatin, and EDC/NHS crosslinked collagen/gelatin/hydroxyethyl cellulose matrices with and without microspheres.

Sample	Position of the Band (cm^-1^)						
	Amide A	Amide B	C–H	Amide I	Amide II	Amide III	C–O
col	3419	3077	2924	1657	1553	1241	1057
gel	3291	3067	2925	1623	1520	1225	1063
col/gel/hec	3294	3064	2922	1631	1542	1226	1054
col/gel/hec (gel)	3296	3066	2919	1627	1535	1232	1056
col/gel/hec (col-gel)	3292	3072	2920	1638	1532	1231	1050

**Table 3:** The values of the compressive modulus (*E*_c_) of col/gel/hec—based matrices measured under different conditions.

Sample	*E*_c_ (kPa) ± SD	
	Dry Samples	Samples Soaked in Phosphate Buffer Saline (PBS)
col/gel/hec	7.02 ± 0.09	0.314 ± 0.10
col/gel/hec (gel)	6.97 ± 1.00	0.411 ± 0.08
col/gel/hec (col-gel)	6.44 ± 0.23	0.382 ± 0.07
